# Quercetin upregulates CREM gene expression in cyanide-induced endocrine dysfunction

**DOI:** 10.1016/j.heliyon.2021.e06901

**Published:** 2021-05-06

**Authors:** Adeoye Oyewopo, Opeyemi Adeleke, Olawumi Johnson, Adebanji Akingbade

**Affiliations:** a Department of Anatomy, College of Health Sciences, University of Ilorin, Ilorin, Kwara State, Nigeria; b Department of Anatomy, College of Health Sciences, Osun State University, Osogbo, Osun State, Nigeria; c Department of Anatomy, University of Medical Sciences, Ondo City, Ondo State, Nigeria; d Department of Anatomy, College of Medicine and Health Sciences, Ekiti State University, Ekiti State, Nigeria

**Keywords:** CREM gene, Cyanide, Quercetin, Testicular toxicity, Testosterone

## Abstract

Cyanide is among the ubiquitous chemicals that humans are usually exposed to and it is well documented that cyanide induces infertility in humans and experimental rodents. However, the pathogenesis remains unknown. Likewise, quercetin is an important nutraceutical that detoxifies reactive oxygen species, but its effects on testicular damage is not clear. The present study investigated the role of nutraceutical, quercetin on cyanide-induced testicular toxicity and probable involvement of cAMP-response-element modulator (CREM) which is a transcription factor necessary for the process of spermatogenesis. Thus, this work hypothesized that quercetin will mitigate endocrine dysfunction induced by cyanide. Seventy-two adult male Wistar rats were divided into seven groups (A to G). Groups A, B, C, F and G comprised of eight (8) rats per group while groups D and E comprised of sixteen (16) rats per group. Group A was designated as control while Groups B and C were given 0.5 and 1 mg/kg of cyanide respectively for 56 days. Group D and E received 0.5 and 1 mg/kg body weight cyanide respectively for 30 days. At day 30, eight animals were sacrificed from Group D and E and the remaining eight (8) rats were subdivided into sub-groups (D1 and E1) and were given 20 and 40 mg/kg of quercetin respectively for twenty-six (26) days. Group F and G were given concurrent administration of cyanide and quercetin at a dose of 0.5 + 20 mg/kg and 1 + 40 mg/kg respectively for 56 days. Body and testicular weight were significantly reduced in cyanide treated groups while quercetin modulates the reduction. Significant down-regulation of CREM gene and reduction in serum level of follicle stimulating hormone (FSH), Luteinizing hormone (LH), testosterone, glutathione peroxidase (GPx) and zinc in cyanide-treated groups, whereas administration of quercetin concomitantly with cyanide exposure or post-treated significantly reversed the alterations.

## 1 Introduction

Previous studies have documented that cyanide induces reproductive and hypothalamic/testicular toxicities (Xavier and William, 2010) and the mechanism underlying these damages are poorly understood. Studies that follow up the morphological changes in human testes have been difficult to carry out due to ethical reasons. However, animal studies with suitable models have been extensively useful. Cyanide (CN) is among the common chemicals that humans are easily exposed to and studies have shown that cyanide induced impaired sperm function and infertility (Shiva et al., 2011) with dearth of information on the possible involvement of cAMP-response-element modulator (CREM) signaling pathways during spermatogenesis. Nevertheless, the extensive exposure of human to chemicals through the water, air, soil and food pollution have been largely attributable to increase number of industries (New York Department of Health, 2020). Among these chemicals is CN, which contains a carbon-nitrogenbond (Environmental and Health Effects of Cyanide, 2020). Its use as a chemical weapon and in agriculture as pesticides and fumigants as well as in plastics, photo developing, mining and electroplating industries (New York Department of Health, 2020). Cyanide can be a life saver as well as deadly poison as earlier report has it that small dose of cyanide in the body system can be converted into a less harmful thiocyanate which is eliminated in urine or combine with hydroxocobalamin to form cyanocobalamin (vitamin B-12) that maintains healthy red blood cells and nerves (New York Department of Health, 2020). Exposure to large dose of CN is highly cytotoxic (Shivanoor and David, 2015). Its mode of inducing damage has been associated with excessive reactive oxygen species (ROS) production, in which a decrease in cellular antioxidant capacity results in tissue damage by interrupting the normal cellular signaling pathways (Chandra et al., 2015).

Quercetin is a form of flavonoid group of polyphenols available in a number of fruits, vegetables, leaves and grains. For example, red onions and kale are common foods containing abundance of quercetin (Linus Pauling Institute, 2015). Quercetin is available as a natural products and food supplement used as an anti-inflammatory and an antioxidant. Basic and clinical studies have shown the beneficial potentials of quercetin (Miles et al., 2014). Its potential benefits in cancer and various pathological conditions have been demonstrated (Williams et al., 2004; Murakami et al., 2008; Russo et al., 2014; D'Andrea, 2015). Likewise, earlier studies have shown that quercetin has oestrogenic like activities by activating estrogen receptors, both alpha and beta (Erα Erβ) (Moutsatsou, 2007) with binding IC_50_ values of 1015_n_M and 113_n_M, respectively (Maggiolini et al., 2001; Van der Woude et al., 2005). In fact, in human breast cell lines, quercetin has been https://markerdb.ca/chemicals/1270found to act as an agonist of the G protein-coupled estrogen receptor (GPER) (Maggiolini et al., 2004). Furthermore, studies have shown that administration of quercetin only confers protective effects against various toxic insults (Taslidere et al., 2016; Chen et al., 2016; Rezaei-Sadabady et al., 2016). However, its effects on male infertility are not clear.

CREM is a member of the basic domain-leucine zipper class of transcription factor which bind as homo and heterodimers to a regulatory palindromic DNA sequence, the cAMP response element (CRE). CRE is localized in the promoter regions of cAMP responsive genes. The absence of CREM-dependent transcription in post-meiotic germ cells results in an arrest of spermatid differentiation with consequent apoptosis (Hogeveen and Sassone-Coris, 2006). Several spermatid-specific genes are known to contain CRE serving as binding site for the transcription factor CREM (Sassone-Corsi, 1995). CREM is essential for spermatogenesis and previous studies have shown that the absence of functional CREM gene caused impaired spermatid maturation with consequent sterility (Blendy et al., 1996; Nantel et al., 1996). In addition, some other studies reported a significant decreased or complete absence of CREM protein (Weinbauer et al., 1998) and CREM mRNA (Steger, 1999) in sterile men. Due to alternative transcriptional start sites, transcript splicing and translational start sites, the CREM gene gives rise to functionally different proteins with either activating or repressing potential on target gene expression (Daniel et al., 2000; Behr and Weinbauer, 2001). However, alterations in CREM expression have been shown to interfere with the spermatid maturation in male (Weinbauer et al., 1998). Nevertheless, the present study was designed to investigate the role of quercetin on CN-induced endocrine dysfunction and probable involvement of CREM. Thus, this work hypothesized that post treatment with quercetin will mitigate endocrine dysfunction induced by cyanide.

In this work, cyanide and quercetin were administered in dose depend manner so as to mimic different degree at which an individual can get exposed to the toxicant and also to establish the treatment regimen of quercetin (either taken quercetin concomitantly when exposed to cyanide or post-treated with quercetin after exposure) that will confer therapeutic effects. This work hypothesized that concomitant administration of quercetin at higher dose will mitigate endocrine dysfunction that may be induced by cyanide.

## 2 Materials and methods

### 2.1 Animal models

Seventy-two (72) nine weeks old male Wistar rats weighing between 180–200 g were procured from the central animal house of college of health sciences, University of Ilorin, bred and housed in the animal house. The animals were maintained in the animal room of the Department of Anatomy under standard laboratory conditions of temperature 27–30 °C. The animals were fed with flavonoid free pelletized feed (grower mash) with access to drinking water *ad libitum.* After acclimatizing for two weeks, the rats were divided into seven groups (A to G). Groups A, B, C, F and G comprised of eight (8) rats per group while groups D and E comprised of sixteen (16) rats per group. Group A (control) received normal saline. Group B and C was given 0.5 and 1 mg/kg body weight CN respectively for 56 days while Group D and E received 0.5 and 1 mg/kg body weight CN respectively for 30 days. At day 30, eight animals were sacrificed from Group D and E and the remaining eight (8) rats were subdivided into sub-groups (D1 and E1) and were given 20 and 40 mg/kg body weight of quercetin respectively for only twenty-six (26) days. Group F and G were given concurrent administration of cyanide and quercetin at a dose of 0.5 mg/kg CN+20 mg/kg quercetin and 1 mg/kg CN+40 mg/kg quercetin respectively for 56 days. Ethical approval with approval number (UERC/ASN/2017/1067) was gotten from University of Ilorin ethical review committee (UERC) and all the experimental procedures were done following the experimental guidelines of Institutional Ethical Review Committee of the University of Ilorin, Kwara State.

### 2.2 Materials

#### 2.2.1 Cyanide and quercetin preparation

Sodium cyanide (≥95% purity; CAS number: 143-33-9) and Quercetin (≥95% purity; CAS number: 117-39-5) were procured from Sigma–Aldrich Chemical Co., USA and authenticated at Pharmacy Department, University of Ilorin, Kwara State. 100 mg of Cyanide was dissolved in 100ml of distilled water while 1 g of Quercetin was dissolved in 100 ml of dimethyl sulfoxide. The solution were allow to stand for some minutes with constant shaken for proper dissolution.

### 2.3 Animal sacrifies and samples collection

The animals were sacrifice on the 57th days of the experiment by anaesthetizing the animals with 80 mg/kg body weight of ketamine and fixed by transcardial perfusion method using 4% paraformaldehyde as fixative. The animals blood was drawn from the left ventricle prior to transcardial perfusion and appropriately dispensed into heparinized bottles for serum levels of prolactin (PRL), testosterone, follicle stimulating hormone (FSH) and luteinizing hormone (LH) analysis. Small testicular tissues were excised prior to perfusion fixation for reverse transcriptase polymerises chain reaction (RT-PCR) analysis.

### 2.4 Oxidative stress biomarkers

The testicular tissue samples were homogenized in 0.1 M phosphate buffer (pH 7.4) using a Potter Elvhjem homogenizer. Lysates from the testes were centrifuged for 10 min in a microfuge at 12,000 rpm to obtain the supernatant containing organelle fragments and synaptosomes and the supernatant fractions were obtained for various biochemical parameters which included estimation of glutathione peroxidase (Gpx) and superoxide dismutase (SOD) concentration. The absorbance of the clear supernatant was measured spectrophotometrically.

### 2.5 Hormone measuring assay

Blood was collected by cardiac puncture into a heparinized bottle and serum was immediately collected by centrifugation (4000 rpm at 4 °C) and stored at -20 °C before the specimen was assayed. All ELISA kits (LH kit with product code: 625-300; PRL kit with product code: 725-300; FSH kit with product code: 425-300 and Testosterone kit with product code: 3725-300) used for this analysis was procured from Monobind Inc. Lake Forest, CA 92630, USA. The essential reagents used for this assay were biotinylated antibody, enzyme-antigen conjugate and a serum native antigen, upon mixing all these reagents, a competitive reaction set-in between the native antigen and enzyme antigen conjugate for a limited number of antibody binding sites. The amount of LH, PRL, FSH and testosterone that were able to bind to the LH, PRL, FSH and testosterone antiserum were inversely proportional to the concentration of LH, PRL, FSH and testosterone in the wells. The absorbance in each well at 450nm was read in a microplate reader (using a reference wavelength of 620–630 nm to minimize well imperfections).

### 2.6 Reverse transcriptase polymerase chain reaction analysis

RNA was isolated from testis using TRIzol Reagent (ThermoFisher Scientific). Purified DNA-free RNA was converted to cDNA immediately using ProtoScript ® First Strand cDNA Synthesis Kit (NEB). Total cDNA (5 uL, 10 ng) was subjected to PCR amplification in a 50 uL reaction mixture containing 10 uL PCR buffer (10 mM Tris–HCl, pH 8.4, 50 mM KCl/1.5 mM MgCl2), 2.5 uL (10 mM) each deoxynucleotide triphosphate, 5 uL each of forward and reverse (10 mM) primers. Amplification conditions were: Pre-denaturation at 94°C for 5 min, Denaturation at 94°C for 30 s, Annealling at 58°C for 30 s and Extension at 72°C for 30 s then 5 min at 72°C by 30 cycles. The amplicons generated during the PCR step was resolved on 5% Agarose gel. In-gel expression bands were captured using iPhone-5c camera (Noir effect). Gel image post-processing was done on Keynote platform on MacBook Pro iOS computer. The densitometric analysis was done using Image-J software (2.3.0 V, Mac version) and finally the bar chart showing the relative expression of target genes was done on Graphpad Prism platform (version 8.03, for Mac iOS).

### 2.7 Statistical analysis

GraphPad Prism version 7.0 was used for the statistical analyses. All data were expressed as mean ± SD. Differences among the groups were analyzed by one-way ANOVA while Bonferroni correction was used to adjust for multiple comparisons. P value < 0.05 was considered to be statistically significant.

## 3 Results

### 3.1 Quercetin improves body and testicular weight loss induced by cyanide

The body weights of the animals were taken on the first day of administration and the day of sacrifice. The weight gain was estimated as the difference between the initial weight of the animal and the final weight.

**Figure 1:**
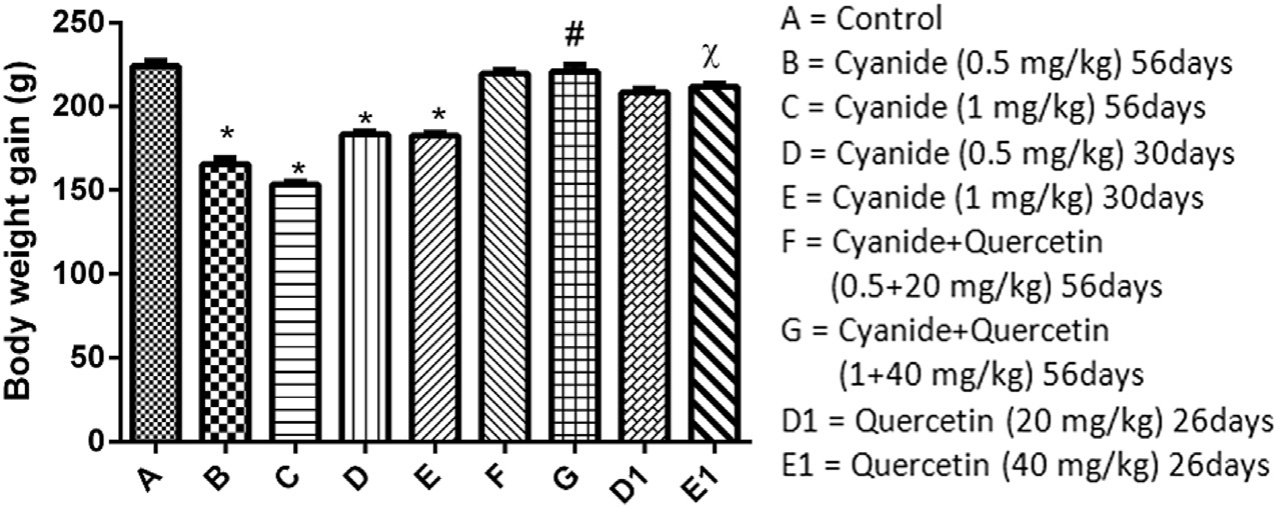
Showed comparison in animals' body weight gained among the groups after the administration of Cyanide and Quercetin. ∗P < 0.05; (n = 8).∗ = is the significant level of difference in comparison with Control Group A; # = is the significant level of difference when groups E and E1 were compared with Group G, χ = is the significant level of difference when group C was compared with group E1.

Figure 1 revealed a decreased in body weight gain in animals treated with cyanide only (groups B to E) which was significant when compared with the control group (p < 0.05). The observed decrease was influenced by the dose of cyanide administered and duration of treatment. Animals post-treated with Quercetin (groups D1 and E1) had an increased in body weight gain which was significant when compared with animals treated with cyanide only. There was also a positive weight gain observed in animals that received concomitant administration of cyanide and quercetin (groups F and G) compared with groups post treated with quercetin (D1 and E1) (though not scientifically significant in dose dependent paradigm).

**Figure 2:**
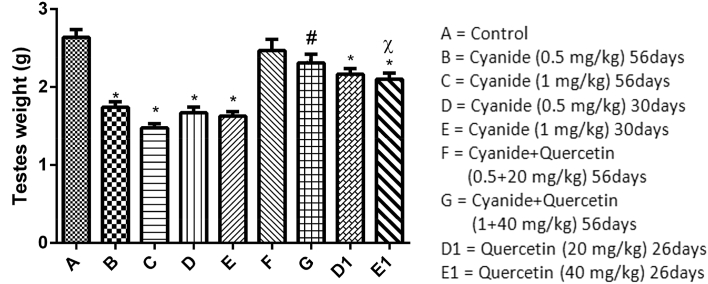
Showed comparison in animals' testicular weight among the groups after the administration of Cyanide and Quercetin. ∗P < 0.05; (n = 8).∗ = is the significant level of difference in comparison with Control Group A; # = is the significant level of difference when groups E and E1 were compared with Group G, χ = is the significant level of difference when group C was compared with group E1.

From Figure 2, a decreased in testes weight was seen in animals treated with cyanide only (groups B to E) which was significant when compared with the control group (p < 0.05). The observed decrease was influenced by the dose of cyanide administered and duration of treatment. Animals post-treated with Quercetin (groups D1 and E1) had an increased testes weight which was significant when compared with animals treated with cyanide only. However, there was a significant decrease in the testes weight in animals pre and post-treated with 1 mg/kg of cyanide (group E) and 40 mg/kg of quercetin (group E1) respectively when compared with the control group. Increased values of testes weight was also recorded in animals that received concomitant administration of cyanide and quercetin (groups F and G) when compared with groups post treated with quercetin (D1 and E1).

### 3.2 Quercetin increased serum GPx and SOD concentration decreased by cyanide

**Figure 3:**
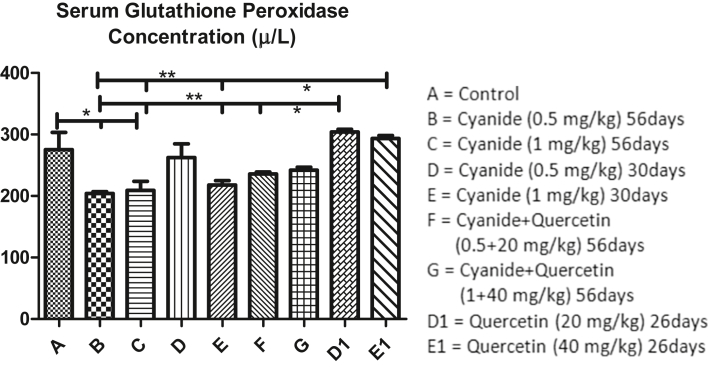
Showed comparison in Serum Glutathione Peroxidase Concentration among the groups after the administration of Cyanide and Quercetin. ∗P < 0.05; ∗∗P < 0.01; (n = 8).

Figure 3 revealed mean values of serum glutathione peroxidase concentration after administration of cyanide and quercetin. Serum glutathione peroxidase concentration was slightly decreased significantly in cyanide treated groups (B and C) when compared with the control group A. Furthermore, slight significant decreased was observed in cyanide treated group B, C and E when compared with quercetin only treated groups D1 and E1. No significant difference was observed among control group A, cyanide treated group D, co-administration of cyanide and quercetin treated group G and quercetin only treated groups D1 and E1.

Group D1 post treated with quercetin for 26 days had slight significant increased in Gpx concentration when compared with group F where quercetin was co-administered with cyanide for 56 days.

**Figure 4:**
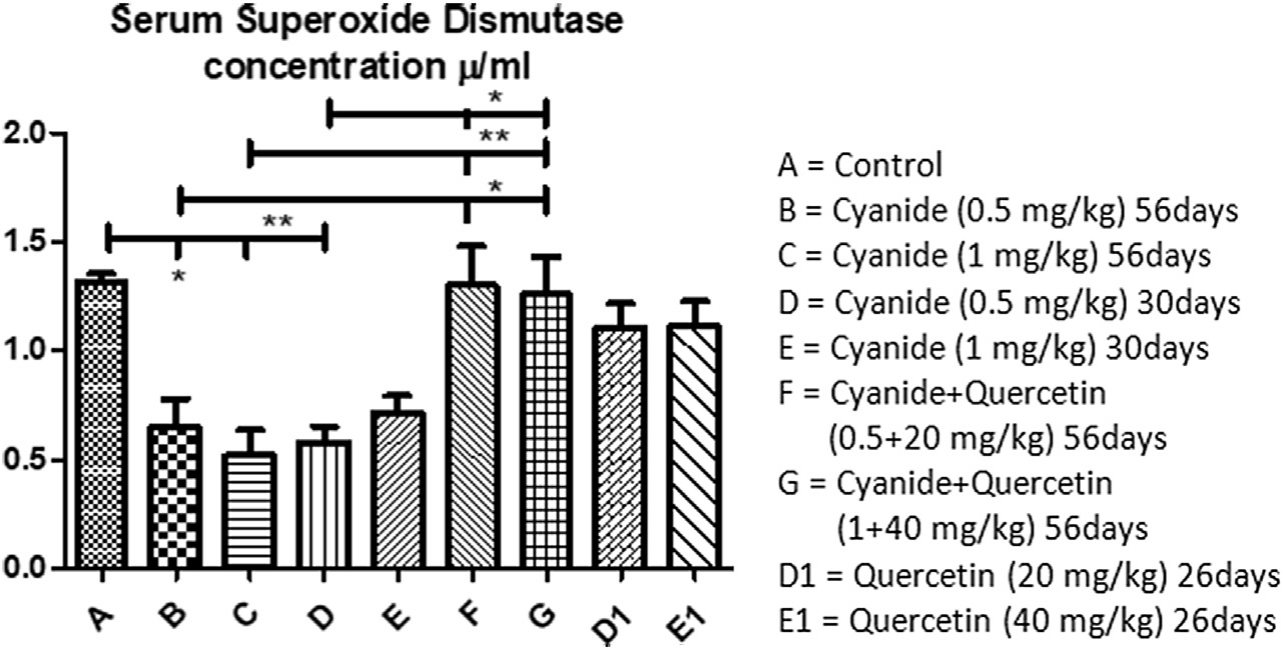
Showed comparison in Serum superoxide dismutase concentration among the groups after the administration of Cyanide and Quercetin. ∗P < 0.05; ∗∗P < 0.01; (n = 8).

Mean values of serum superoxide dismutase concentration among experimental groups has revealed in Figure 4 showed significant decreased of superoxide dismutase concentration in cyanide treated groups (B, C and D) when compared with control group A; groups F and G treated with cyanide and quercetin concomitantly. No significant difference were seen among the control group A, co-administration of cyanide and quercetin treated groups F and G and quercetin only post-treated groups D1 and E1.

More also, increased in serum superoxide dismutase concentration was noted in groups (F and G) that concomitantly given cyanide and quercetin when compared with groups (D1 and E1) post-treated with quercetin for 26 days after 30 days cyanide exposure (though not scientifically significant).

### 3.3 Quercetin normalized serum hormonal concentration disturbed by cyanide

**Figure 5:**
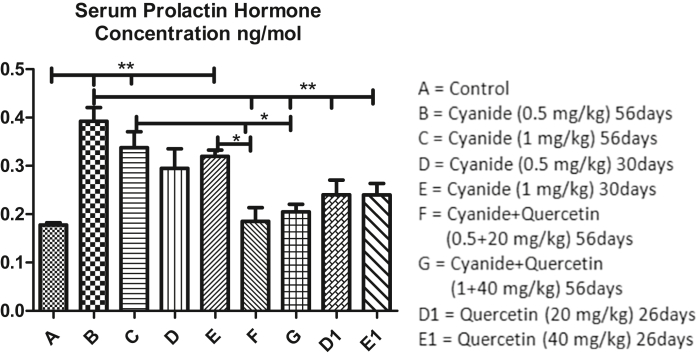
Showed comparison in Serum Prolactin hormone Concentration among the groups after the administration of Cyanide and Quercetin.∗P < 0.05; ∗∗P < 0.01; (n = 8).

Mean values of serum prolactin hormone among experimental groups has depicted in Figure 5 showed high significant increased in cyanide treated groups (B and C) and slight significant increased in cyanide treated group E when compared with the control group A. Moreover, high significant increased in serum prolactin hormone concentration was seen in cyanide treated group B when compared with co-administration of cyanide and quercetin (F and G) and quercetin only treated groups (D1 and E1).

Furthermore, comparison among cyanide treated group C and co-administration of cyanide and quercetin treated groups F and G showed slight significant increased of prolactin in cyanide treated group C when compared with co-administration of cyanide and quercetin treated groups F and G. Also slight significant increased in prolactin concentration was observed in cyanide treated group E when compared with co-administration of cyanide and quercetin treated groups F while no significant difference was seen when compared quercetin only treated groups D1 and E1 with cyanide treated groups C, D and E; co-administration of cyanide and quercetin treated groups F and G and control group A.

Groups D and E that were exposed to cyanide for 30 days had lesser prolactin concentration when compared with groups B and C that were exposed to cyanide for 56 days. Groups D1 and E1 post treated with quercetin for 26 days also had decreased in serum prolactin concentration when compared with groups F and G where quercetin was co-administered with cyanide for 56 days (though not scientifically significant in dose dependent paradigm).

**Figure 6:**
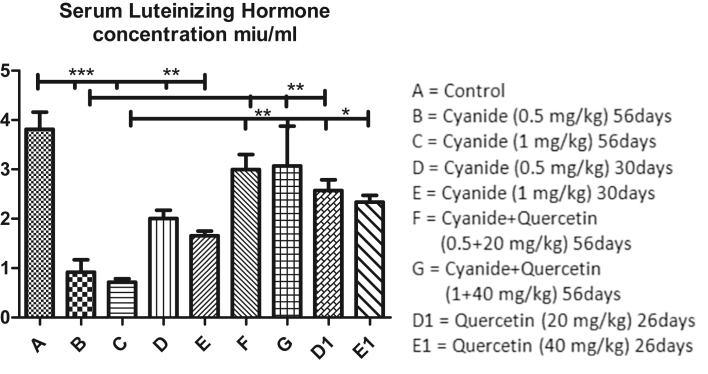
Showed comparison in Serum Luteinizing hormone Concentration among the groups after the administration of Cyanide and Quercetin. ∗P < 0.05; ∗∗P < 0.01; ∗∗∗P < 0.001 (n = 8).

Figure 6 graph revealed mean values of serum luteinizing hormone concentration among the groups after the administration of cyanide and quercetin. Serum luteinizing hormone concentration were significantly decreased in all cyanide treated groups (B, C, D and E) when compared with the control group A and co-administration of cyanide and quercetin (F and G) treated groups. Moreover, no significant difference was seen among control group A, co-administration of cyanide and quercetin treated groups F and G and quercetin only treated groups D1 and E1.

Increased in serum luteinizing hormone concentration in dose dependent manner was also observed in cyanide only treated groups (D and E) for 30 days when compared with groups treated with cyanide only for 56 days (groups B and C) (though the differences were not scientifically significant).

**Figure 7:**
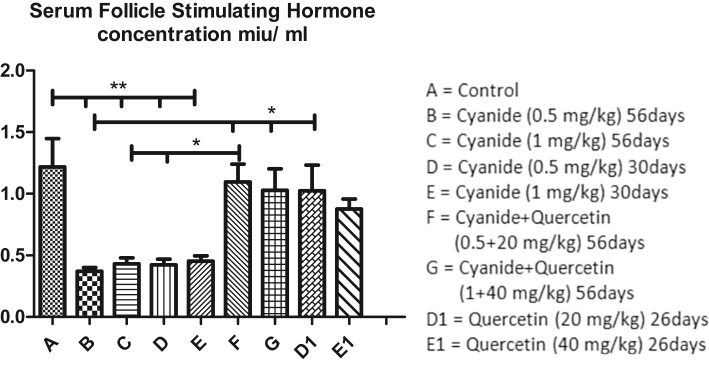
Showed comparison in Serum Follicle Stimulating hormone Concentration among the groups after the administration of Cyanide and Quercetin. ∗P < 0.05; ∗∗P < 0.01; (n = 8).

Figure 7 revealed mean values of serum follicle stimulating hormone concentration after administration of cyanide and quercetin. Serum follicle stimulating hormone concentration was significantly decreased in cyanide treated groups (B, C, D and E) when compared with the control group A. Furthermore, slight significant decreased in serum follicle stimulating hormone Concentration was observed in cyanide treated group B when compared with co-administration of cyanide and quercetin (F and G) and quercetin only treated group D1. Cyanide treated groups C and D showed slight significant decreased in follicle stimulating hormone concentration when compared with co-administration of cyanide and quercetin treated group F and no significant difference was observed among control group A, co-administration of cyanide and quercetin treated groups F and G and quercetin only treated groups D1 and E1.

**Figure 8:**
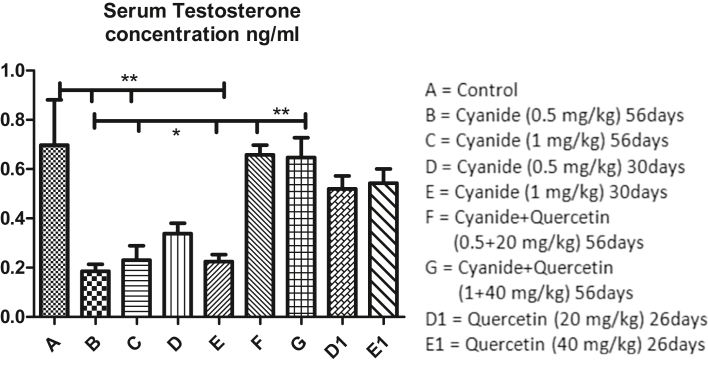
Showed comparison in Serum Testosterone Concentration among the groups after the administration of Cyanide and Quercetin. ∗P < 0.05; ∗∗P < 0.01; (n = 8).

Mean values of serum testosterone hormone concentration as depicted in Figure 8 showed significant decreased in cyanide treated groups (B, C and E) when compared with the control group A. Moreover, slight significant decreased in serum testosterone hormone Concentration was observed in cyanide treated groups B, C and E when compared with co-administration of cyanide and quercetin (F and G) treated groups. No significant difference was seen among control group A, cyanide treated group D, co-administration of cyanide and quercetin treated groups F and G and quercetin only treated groups D1 and E1.

Increased in serum testosterone hormone concentration in dose dependent manner was also observed in cyanide only treated groups (D and E) for 30 days when compared with groups treated with cyanide only for 56 days (groups B and C) (though the differences were not scientifically significant).

### 3.4 Quercetin up-regulate CREM gene expression down-regulated by cyanide

**Figure 9:**
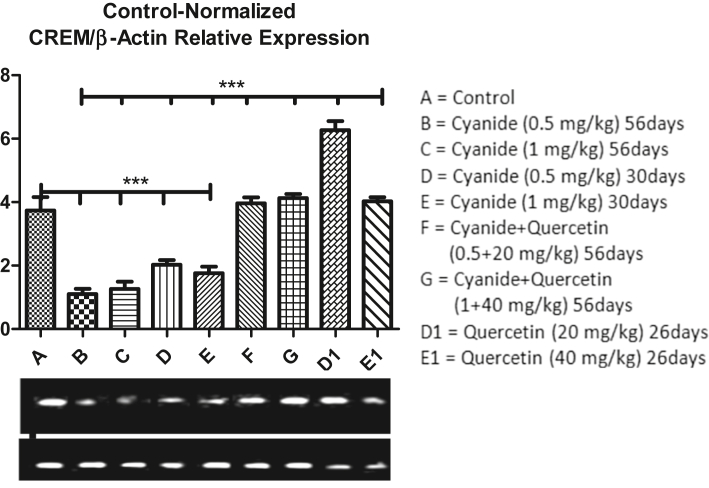
Showed comparison in CREM gene expression among the groups after the administration of Cyanide and Quercetin. ∗∗∗P < 0.001 (n = 8).

Result from Figure 9 revealed CREM gene expression mean values after the administration of Cyanide and Quercetin. High significant down-regulation of CREM gene was observed in all cyanide treated groups (B, C, D and E) when compared with control group A whereas, high significant up-regulation of CREM gene expression was observed in quercetin only treated group D1 when compared with control group A rats. Comparison in CREM gene expression among cyanide treated groups (B, C, D and E), Co-administration of cyanide and quercetin treated groups (F and G) and quercetin treated groups only (D1 and E1) showed high significant up-regulation of CREM gene expression in Co-administration of cyanide and quercetin treated groups and quercetin treated groups only when compared with cyanide only treated groups. Furthermore, Significant up-regulation of CREM gene were observed in quercetin only treated group D1 when compare with co-administration of cyanide and quercetin treated groups F and G. Expression of CREM gene in dose dependent manner was slightly higher in cyanide only treated groups (D and E) for 30 days when compared with groups treated with cyanide only for 56 days (groups B and C) (though the differences were not scientifically significant).

## 4 Discussion

This research work demonstrates the efficacy of nutraceutical (quercetin) in attenuating testicular toxicity induced by cyanide. The morphometric data obtained from this study indicate that the animals’ body weight and testicular weight were significant reduced in cyanide treated groups while cyanide co-administered with quercetin treated groups showed significant weight gain. This result is in line with Sanni et al. (2020) who reported significant weight loss in rats treated with cyanide and also with Rezaei-Sadabady et al. (2016)paper where they reported that quercetin prevents body weight loss due to the using of superparamagnetic iron oxide nanoparticles in rat. The decrease in body weight observed in the cyanide treated group might be due to the loss of appetite as a result of gastrointestinal tract irritation. Tulsawani et al. (2005); Bolaji and Olabode (2011) reported that oral administration of potassium cyanide result in decreased water and food consumption by rats and mice. This suggests poor palatability (Tulsawani et al., 2005). Furthermore, quercetin reversed the weight and testicular lost in cyanide co-administered with quercetin treated groups.

The results of this study showed significant down regulation of CREM gene expression in all cyanide treated groups when compared with the control group. Furthermore, serum LH, FSH and testosterone concentration were significantly decreased in cyanide treated groups while prolactin level was significantly increased. This results were in line with Muniswamy and Shiddapa who reported sperm abnormalities and significant decreased in the levels of LH, FSH and testosterone hormone in male wistar albino rats after administration of cyanide (Muniswamy and Shiddapa, 2015).

Reproductive toxicity induced by cyanide as indicated above might be as a result of cyanide preventing specialized nerve cells in the hypothalamus and gonadotropic cells in the adenohypophysis from using oxygen to make energy molecules as previously reported (Sassone-Corsi, 1998). Thereby subjecting the nerve cell to necrosis and apoptosis through oxidative stress, therefore pulsative production of GnRH hormones is altered and its resultant effects affect FSH and LH production in the adenohypophysis. Furthermore, FSH has been reported to be a key player in the transcription process of CREM gene, absence of CREM -dependent transcription in post-meiotic germ cells results in an arrest of spermatid differentiation and apoptosis (Sassone-Corsi, 1998; Wang et al., 2018). Significant up regulation of CREM gene and increased in LH, FSH, testosterone, glutathione peroxidase, superoxide dismutase concentration were observed in the cyanide and quercetin treated groups and quercetin only treated groups while prolactin concentration were the same with the control group.

However, serum glutathione peroxidase and superoxide dismutase concentration were found to be significantly reduced in cyanide treated groups while groups concomitantly administered with both cyanide and quercetin for 56 days and the groups post-treated with quercetin for 26 days after 30 days exposure to cyanide showed significant increased in glutathione peroxidase and superoxide dismutase concentration. This implies that either taken quercetin concomitantly after being exposed to cyanide or take it after the exposure proved to be remedy to any form of endocrine dysfunction that may induced by cyanide exposure (though from this findings, taken quercetin concomitantly when exposed to cyanide proved to be the best remedy). This reports were in accord with Richard and Howard study where they reported the glutathione peroxidase inhibition by cyanide (Richard and Howard, 1980). Moreso, Okafor et al. (2006) reported significant depletion in whole blood glutathione concentration after the administration of cassava cyanide to rats.

**Figure 10:**
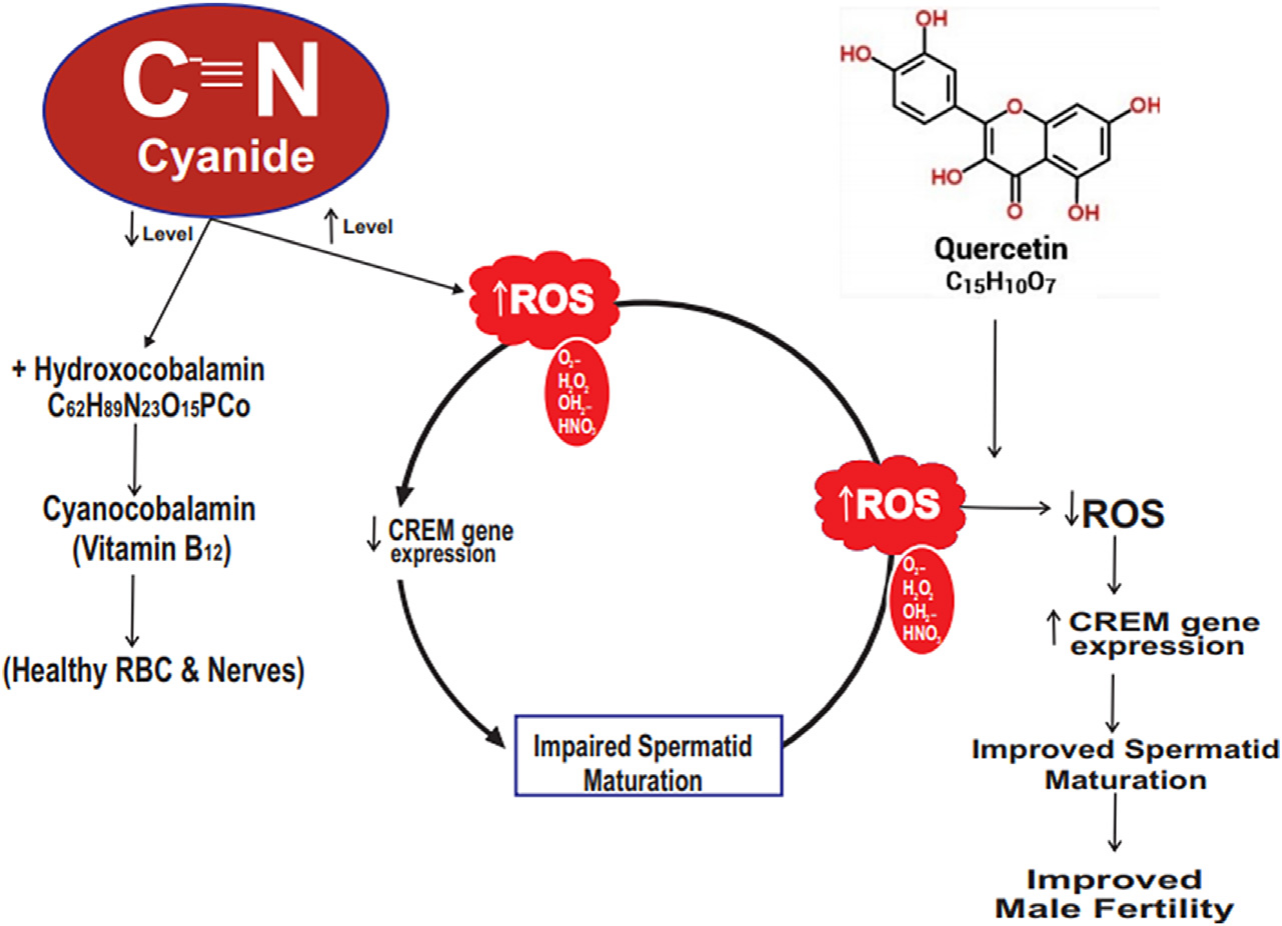
Schematic diagram showing the pathway of quercetin inhibiting testicular toxicity induced by Cyanide.

Furthermore, Changwon et al. (2020) reported that quercetin up regulates spermatogenesis related genes of mouse exposed to high cholesterol diet. In the case of ameliorating effects observed in quercetin treated groups might be as a result of polyphenolic chemical substructure found in quercetin. As shown in Figure 10, when systemic metal ion balance is disrupted, there will be disequilibrium between the ROS and cellular anti-oxidants resulting to damage which may include protein modification, DNA damage, lipid peroxidation among others (Klaudia and Marian, 2011; Aitken and Roman, 2013). Although the testicular microenvironment has low oxygen tensions, testis remains vulnerable to oxidative stress due to the abundance of highly unsaturated fatty acids (Wu et al., 2016), and quercetin in this study was found to reverse the oxidative chain reactions by up-regulating the expression of CREM gene, increased LH, FSH, testosterone, glutathione peroxidase, superoxide dismutase concentration, thus ameliorated endocrine dysfunction in cyanide-treated animals.

## 5 Conclusion

The present study reveals that cyanide-induced endocrine dysfunction is attributable to down-regulation of CREM gene expression and decreased in selected endocrine hormones (FSH, LH and testosterone). The study in addition suggests the ameliorative effects of quercetin on endocrine dysfunction by improving the expression of CREM and endocrine hormones. While post treating cyanide toxicity by quercetin proved effective in this study, taken quercetin-rich supplements concomitantly when someone is exposed to cyanide is the best therapeutic way of mitigating the endocrine dysfunction that maybe induced by cyanide.

## Declarations

### Author contribution statement

Adeoye Oyewopo: Conceived and designed the experiments; Performed the experiments; Analyzed and interpreted the data; Contributed reagents, materials, analysis tools or data; Wrote the paper.

Opeyemi Adeleke: Contributed reagents, materials, analysis tools or data.

Olawumi Johnson, Adebanji Akingbade: Performed the experiments; Contributed reagents, materials, analysis tools or data.

### Funding statement

This work was supported by the Tertiary education trusts fund (2017–2018 IBR/TETFUND/DESS/UNI/ILORIN/RP/VOL.IV)under its Institution-based Research Grant (IBR).

### Data availability statement

Data will be made available on request.

### Declaration of interests statement

The authors declare no conflict of interest.

### Additional information

No additional information is available for this paper.
